# Soft Tissue Augmentation Decision-Making, Failure Attribution, and Outcome Calibration in Implant Dentistry: A Cross-Sectional Survey

**DOI:** 10.7759/cureus.109202

**Published:** 2026-05-19

**Authors:** Priyanka Vhanmane, Vidya Dodwad, Banashree Sankeshwari, Yogesh Khadtare, Krishna Patil, Purva Chougule

**Affiliations:** 1 Department of Periodontology, Bharati Vidyapeeth (Deemed to be University) Dental College and Hospital, Sangli, IND; 2 Department of Periodontology, Bharati Vidyapeeth (Deemed to be University) Dental College and Hospital, Pune, IND; 3 Department of Prosthodontics, Bharati Vidyapeeth (Deemed to be University) Dental College and Hospital, Sangli, IND; 4 Department of Pedodontics and Preventive Dentistry, Bharati Vidyapeeth (Deemed to be University) Dental College and Hospital, Pune, IND

**Keywords:** connective tissue graft, dental implants, keratinized tissue, soft tissue augmentation, treatment outcome

## Abstract

Introduction: Soft-tissue augmentation around dental implants plays a critical role in ensuring peri-implant health, esthetic outcomes, and long-term stability. However, variability exists in clinical decision making, technique selection, and outcome evaluation among practitioners. This study aimed to assess decision-making patterns, perceived failure rates, and outcome evaluations related to soft tissue augmentation in implant dentistry.

Materials and methods: A cross-sectional, questionnaire-based study was conducted among 150 clinicians involved in implant therapy, including periodontists, oral surgeons, and prosthodontists. Participants were recruited through professional networks using a convenience sampling approach. A structured and validated questionnaire assessed demographics, clinical decision thresholds, technique preferences, failure attribution, outcome perception, clinician confidence, and complications using multiple-choice, scenario-based, and Likert scale items. Data were collected electronically and analyzed using descriptive and inferential statistics, with significance set at p < 0.05.

Results: Among the participants, 95 (63.3%) were males, and 55 (36.7%) were females. Most clinicians have reported 11-15 years of experience and placed 11-20 implants per month. The majority preferred augmentation at the 1-2 mm keratinized tissue level across specialties. Connective tissue grafting was the preferred technique (p = 0.027). Most respondents estimated failure rates at 5-10% and favored shared responsibility in the case of failure. Excellent outcomes are widely accepted as successful, whereas moderate outcomes are less frequently considered publishable. Confidence in connective tissue grafting was higher among males (3.43 ± 0.79 vs. 3.13 ± 0.90, p = 0.041), while confidence in biomaterials varied across specialties (p = 0.004). Despite the clinical success, patient dissatisfaction was the most common complication.

Conclusions: Considerable variability exists in clinical decision-making, technique selection, and outcome perception in soft tissue augmentation. Although clinicians show general agreement on intervention thresholds, differences in confidence and failure attribution persist. The gap between clinically acceptable and publishable outcomes highlights the subjective nature of success. Standardized guidelines, structured training, and improved patient communication are essential to enhance consistency and optimize outcomes.

## Introduction

The paradigm of implant dentistry has evolved beyond mere osseointegration to encompass the preservation and optimization of peri-implant soft tissues, which are critical for long-term functional stability and esthetic success [1]. Adequate keratinized tissue width and sufficient mucosal thickness are closely linked to improved plaque control, decreased peri-implant inflammation, and greater patient satisfaction [2]. Consequently, these soft tissue parameters have gained increasing importance because of their significant impact on prosthetic outcomes and long-term peri-implant tissue stability [3]. Consequently, soft tissue augmentation procedures such as connective tissue grafts, free gingival grafts, and the use of xenogeneic collagen matrices are increasingly incorporated into routine implant therapy [4]. However, there remains a lack of consensus regarding the precise clinical thresholds that necessitate intervention, leading to significant variability in practice patterns. Additionally, patient-related behavioral factors may further influence treatment planning decisions in implant therapy [5].

Clinical decision-making in soft tissue augmentation is multifactorial and often influenced by clinician experience, specialty training, case complexity, and patient-related risk factors, such as smoking and oral hygiene status [6]. Moreover, while objective parameters such as keratinized tissue width and soft tissue thickness are measurable, interpretation of what constitutes a “successful” outcome varies considerably among clinicians. This discrepancy is particularly evident when comparing outcomes deemed acceptable in daily practice to those considered suitable for academic publication. In addition, failure in soft tissue augmentation, often defined by inadequate tissue gain and graft failure as one of the complications, raises important questions regarding responsibility, cost burden, and medico-legal implications, areas that remain insufficiently explored in the current literature [7,8].

Given these gaps, there is a need to systematically evaluate how clinicians approach decision-making, perceive outcomes, and attribute responsibility in the context of soft tissue augmentation around dental implants. This study aimed to assess decision-making patterns, perceived failure rates, and outcome evaluations in soft tissue augmentation in implant dentistry. The objectives were to analyze the clinical thresholds for augmentation, evaluate technique preferences in different scenarios, assess perceptions of failure and cost attribution, compare subjective and objective success criteria, and examine clinician confidence, training background, and reported complications.

## Materials and methods

Study design and setting

This cross-sectional, questionnaire-based study was conducted in the Department of Periodontology at Bharati Vidyapeeth (Deemed to be University) Dental College and Hospital, Sangli, India, over a period of three months, from January 2025 to March 2025. This study was designed to explore clinical decision-making patterns, outcome assessments, and failure attributions related to soft tissue augmentation procedures in implant dentistry.

Ethical considerations

Ethical clearance was obtained from the institutional ethics committee (Ref no-BVDU/DCH//09/2024-25) before the commencement of the study. This study was conducted in accordance with the principles of the Declaration of Helsinki. Participation was voluntary, and informed consent was obtained from all participants before they completed the questionnaire. The anonymity of the respondents was preserved, and all data were handled with strict confidentiality.

Sample size justification

The sample size was determined using a pragmatic approach due to the exploratory nature of the study. As no prior data were available for effect size estimation across all questionnaire domains, a formal power calculation was not feasible. Therefore, a minimum target sample of 120 participants was considered adequate based on similar cross-sectional surveys in implant dentistry [9]. A total of 150 responses were ultimately included, which exceeded this threshold and was deemed sufficient to provide stable estimates for descriptive and inferential analyses.

Study population and sampling

The study population consisted of 150 dental professionals involved in implant therapy, including periodontists, oral surgeons, and prosthodontists. Participants were recruited using a non-probability convenience sampling method using professional networks, institutional contacts, and digital platforms. This approach may introduce selection bias and limit generalizability; however, it was considered appropriate for exploratory assessment of clinician perspectives. Clinicians with active involvement in implant placement or restoration and a minimum of one year of clinical experience were included in the study. The participants primarily consisted of qualified dental specialists and practicing clinicians actively involved in implant placement or restoration procedures, whereas postgraduate residents were not recruited as a separate study subgroup. Responses that were incomplete or submitted by individuals not engaged in implant practice were excluded from analysis.

Questionnaire design and validation

A structured, self-administered questionnaire was developed based on the relevant literature and clinical expertise to assess various aspects of soft tissue augmentation (Appendix 1). The questionnaire comprised five domains: demographic and practice characteristics; clinical decision thresholds; failure attribution and economic considerations; subjective versus objective outcome evaluation using clinical scenarios; and clinician confidence, training, and complications. The questionnaire included multiple-choice items, scenario-based questions, and Likert scale responses. Content validity of the questionnaire was assessed by a panel of five subject experts in periodontology and implant dentistry. Each item was evaluated for relevance, clarity, and comprehensiveness. A pilot study was conducted on 15 clinicians to assess feasibility and clarity, and minor modifications were made accordingly. Internal consistency of Likert-scale items was evaluated using Cronbach’s alpha (α = 0.82), indicating good reliability.

Data collection procedure

The finalized questionnaire was converted into an electronic format and distributed via email and social media. The participants were invited to complete the survey within the study period, and only one response per participant was permitted. A total of 180 clinicians were invited to participate, of whom 150 completed the questionnaire, yielding a response rate of 83.3%. Responses were automatically recorded and stored in a database. Periodic reminders were sent to improve the response rates.

Outcome measures

The primary outcomes assessed included clinical thresholds for performing soft tissue augmentation, preferred techniques under varying clinical conditions, perceived failure rates, attribution of responsibility for failed procedures, differences between subjective and objective success assessments, and clinician confidence levels, along with reported complications.

Statistical analysis

All data were analyzed using the statistical software, IBM SPSS Statistics for Windows, Version 20 (Released 2018; IBM Corp., Armonk, New York, United States). Categorical variables are expressed as frequencies and percentages. Normality of data was checked with the Shapiro-Wilk test. Associations between categorical variables were evaluated using Pearson’s chi-square test or Fisher’s exact test (if cell value less than 5), as appropriate. Ordinal data derived from Likert-scale responses were analyzed using non-parametric tests. The Mann-Whitney U test was used for comparisons between two groups, and the Kruskal-Wallis H test for comparisons among more than two groups. Where the Kruskal-Wallis test was significant, post hoc pairwise comparisons with Bonferroni correction were performed. Statistical significance was set at p < 0.05.

## Results

A total of 150 responses were analyzed. The study cohort consisted of 95 (63.3%) male participants, with a smaller proportion of females. Periodontists constituted the largest group of specialties, followed by oral surgeons and prosthodontists, indicating a balanced representation of implant-related disciplines. The age distribution revealed that most of the participants belonged to the middle-aged group, reflecting a cohort with substantial clinical exposure. Statistically significant differences were observed in sex, specialty, and age distribution, suggesting a non-uniform but clinically relevant sample composition (p < 0.05) (Table 1).

**Table 1:** Demographic profile of study participants (n = 150). χ²: Pearson chi-square test; *p < 0.05 indicates statistical significance, data are presented as number (percentage), comparisons were performed within each variable to assess distribution differences across categories.

Variable	Category	n (%)	χ²	p-value
Sex	Male	95 (63.3)	10.83	0.001*
	Female	55 (36.7)		
Specialty	Periodontist	65 (43.3)	6.40	0.041*
	Oral Surgeon	45 (30.0)		
	Prosthodontist	40 (26.7)		
Age group	< 30 years	30 (20.0)	8.75	0.013*
	30-45 years	68 (45.3)		
	> 45 years	52 (34.7)		

With respect to clinical experience and workload, the majority of respondents reported moderate to high experience in implant dentistry, with many having more than 10 years of practice. Implant placement frequency varied significantly, with most clinicians placing 11-20 implants per month, followed by those placing 21-30 implants. Similarly, the number of soft tissue augmentation procedures performed annually showed significant variation, with most clinicians performing a moderate number of procedures, whereas fewer reported either very high volumes or no procedures. These findings highlight the variability in clinical exposure and procedural experience among participants (Table 2).

**Table 2:** Practice volume and clinical experience of participants (n = 150). χ²: Pearson chi-square test, *p < 0.05 indicates statistical significance, data are presented as number (percentage), all comparisons were performed within each question to assess distribution across response categories. STA: soft tissue augmentation

Question	Response option	n (%)	χ²	p-value
Q1. Years in implantology	< 5 years	18 (12.0)	12.48	0.006*
	6-10 years	44 (29.3)		
	11-15 years	52 (34.7)		
	> 15 years	36 (24.0)		
Q2. Implants per month	< 10	22 (14.7)	22.96	< 0.001*
	11-20	58 (38.7)		
	21-30	45 (30.0)		
	> 30	25 (16.6)		
Q3. STA procedures/year	None	15 (10.0)	28.30	< 0.001*
	1-5	48 (32.0)		
	6-10	55 (36.7)		
	> 10	32 (21.3)		

The clinical decision-making thresholds for STA demonstrated relative consistency across specialties. Most clinicians prefer to intervene at moderate levels of keratinized tissue deficiency and soft tissue thickness rather than at minimal or severe thresholds. No statistically significant differences were observed among the specialties for these parameters. However, technique selection in a standardized clinical scenario showed significant variation, with connective tissue grafts emerging as the preferred approach across all groups. In contrast, responses regarding modification of the treatment approach in smokers did not differ significantly, with most clinicians opting for more invasive strategies (Table 3).

**Table 3:** Clinical decision thresholds and technique preferences across specialties (n = 150). χ²: Pearson chi-square test, *p < 0.05 indicates statistical significance, data are presented as number (percentage), comparisons performed across specialties. CTG: connective tissue graft, FGG: free gingival graft

Question	Response	Periodontist (n = 65)	Oral Surgeon (n = 45)	Prosthodontist (n = 40)	χ²	p-value
Q4. Minimum keratinized tissue width prompting augmentation	< 1 mm	14 (21.5)	9 (20.0)	5 (12.5)	7.82	0.253
	1-2 mm	28 (43.1)	18 (40.0)	16 (40.0)		
	> 2 mm	16 (24.6)	11 (24.4)	8 (20.0)		
	No augmentation based on keratinized tissue	7 (10.8)	7 (15.6)	11 (27.5)		
Q5. Minimum buccal thickness prompting augmentation	< 1 mm	16 (24.6)	10 (22.2)	6 (15.0)	3.56	0.737
	1-2 mm	25 (38.5)	17 (37.8)	16 (40.0)		
	> 2 mm	12 (18.5)	10 (22.2)	8 (20.0)		
	No augmentation based on thickness	12 (18.5)	8 (17.8)	10 (25.0)		
Q6. Technique choice (thin/translucent mucosa case)	No augmentation	2 (3.1)	4 (8.9)	2 (5.0)	14.28	0.027*
	Xenogeneic collagen matrix	6 (9.2)	8 (17.8)	8 (20.0)		
	CTG	42 (64.6)	24 (53.3)	22 (55.0)		
	FGG	15 (23.1)	9 (20.0)	8 (20.0)		
Q7. Technique modification for heavy smoker	No: same technique	4 (6.2)	4 (8.9)	2 (5.0)	5.11	0.530
	Yes: switch to more invasive	38 (58.5)	24 (53.3)	20 (50.0)		
	Yes: avoid augmentation entirely	14 (21.5)	8 (17.8)	8 (20.0)		
	Yes: refer the patient	9 (13.8)	9 (20.0)	10 (25.0)		

Perceptions regarding failure rates of soft tissue augmentation were largely consistent among respondents, with most estimating a relatively low-to-moderate rate of failure. Attribution of responsibility in cases of unexplained failure revealed a tendency toward shared responsibility between the clinician and the patient. When patient-related risk factors, such as smoking, were introduced, clinicians showed a shift toward increased patient responsibility, although these differences were not statistically significant. Additionally, only a small proportion of participants reported involvement in legal disputes related to augmentation failure, indicating relatively low medicolegal exposure (Table 4).

**Table 4:** Failure attribution and economic responsibility in soft tissue augmentation (n = 150). χ²: Pearson chi-square test, data are presented as number (percentage), comparisons performed between male and female participants, p > 0.05 denotes no statistical significance. STA: soft tissue augmentation, CTG: connective tissue graft

Question	Response	Male (n = 95)	Female (n = 55)	χ²	p-value
Q8. Perceived failure rate of STA	< 5%	28 (29.5)	14 (25.5)	0.49	0.921
	5-10%	42 (44.2)	26 (47.3)		
	11-20%	20 (21.1)	12 (21.8)		
	> 20%	5 (5.3)	3 (5.5)		
Q9. Cost attribution for unexplained CTG failure	Patient bears cost	10 (10.5)	8 (14.5)	1.28	0.734
	Clinician bears cost	18 (18.9)	10 (18.2)		
	Insurance bears cost	20 (21.1)	12 (21.8)		
	Shared 50/50	47 (49.5)	25 (45.5)		
Q10. Cost attribution if the patient is a known smoker	No change	18 (18.9)	10 (18.2)	2.34	0.505
	Patient bears 100%	28 (29.5)	14 (25.5)		
	75% patient / 25% clinician	36 (37.9)	19 (34.5)		
	Clinician bears partial responsibility	13 (13.7)	12 (21.8)		
Q11. Involvement in a legal dispute/complaint	Yes	15 (15.8)	7 (12.7)	0.52	0.470
	No	80 (84.2)	48 (87.3)		

The assessment of treatment outcomes demonstrated a clear gradient in acceptance levels. Excellent clinical outcomes were almost universally considered successful in both daily practice and academic publications. Moderate outcomes were accepted by the majority for routine practice, but were less frequently considered suitable for publication. Poor outcomes were the least accepted across both domains. Importantly, no significant differences were observed among the specialties in the perception of either personal or publishable success, indicating a consistent pattern of outcome evaluation (p > 0.05) (Table 5). Due to the high proportion of affirmative responses (ceiling effect), statistical comparisons should be interpreted cautiously, as limited variability reduces the discriminatory power of inferential tests.

**Table 5:** Subjective and objective outcome assessment across specialties (n = 150). χ²: Pearson chi-square test, data are presented as number (percentage of “Yes” responses), comparisons performed across specialties, p > 0.05 denoted no statistical significance. Scenario 1: poor outcome (<1 mm keratinized tissue, thin mucosa, graft contraction), Scenario 2: moderate outcome (≈2 mm keratinized tissue, ≈1.5 mm thickness), Scenario 3: excellent outcome (>3 mm keratinized tissue, >2 mm thickness).

Criterion	Scenario category	Periodontist, n (%)	Oral Surgeon, n (%)	Prosthodontist, n (%)	χ²	p-value
Q12. Personal success (acceptable in daily practice)	Scenario 1 (Poor outcome)	6 (9.2)	7 (15.6)	5 (12.5)	1.38	0.502
	Scenario 2 (Moderate outcome)	44 (67.7)	32 (71.1)	26 (65.0)	0.43	0.806
	Scenario 3 (Excellent outcome)	63 (96.9)	44 (97.8)	38 (95.0)	0.34	0.844
Q13. Publishable success (acceptable for peer-reviewed reporting)	Scenario 1 (Poor outcome)	2 (3.1)	3 (6.7)	1 (2.5)	1.52	0.468
	Scenario 2 (Moderate outcome)	30 (46.2)	22 (48.9)	16 (40.0)	0.72	0.697
	Scenario 3 (Excellent outcome)	62 (95.4)	42 (93.3)	38 (95.0)	0.22	0.895

Confidence levels in performing soft-tissue augmentation varied across techniques and respondent characteristics. A statistically significant difference was observed in the confidence related to connective tissue graft procedures, with higher confidence levels reported among male clinicians (p < 0.05). Confidence in the use of xenogeneic collagen matrices also differed significantly across specialties, with periodontists demonstrating comparatively higher confidence than the other groups (p = 0.004). These findings suggest that both experience and specialty training influenced perceived competence in the different augmentation techniques (Table 6). Post‑hoc Dunn’s test with Bonferroni correction showed that periodontists (2.80 ± 0.94) had significantly higher scores than oral surgeons (2.53 ± 0.97; adjusted p = 0.0027). No other pairwise differences reached significance.

**Table 6:** Confidence levels in soft tissue augmentation techniques (n = 150). U: Mann-Whitney U test, H: Kruskal-Wallis H test, *p < 0.05 indicates statistical significance, data are presented as mean ± standard deviation, confidence measured on a 4-point Likert scale (1 = not confident, 4 = confident). CTG: connective tissue graft

Variable	Category	Mean ± SD	Mann–Whitney U /Kruskal H	p-value
Q14. Confidence: CTG	Male (n = 95)	3.43 ± 0.79	U = 2388.50	0.041*
	Female (n = 55)	3.13 ± 0.90		
Q15. Confidence in xenogeneic collagen matrix	Periodontist (n = 65)	2.80 ± 0.94	H = 11.24	0.004*
	Oral Surgeon (n = 45)	2.53 ± 0.97		
	Prosthodontist (n = 40)	2.68 ± 0.96		

Regarding complications, patient dissatisfaction despite clinically acceptable outcomes was the most frequently reported issue, followed by graft necrosis and infection, whereas severe postoperative bleeding was less common. No significant differences were observed across specialties in complication rates, indicating a broadly similar clinical experience (p > 0.05). In contrast, the source of training showed significant variation, with private training being the most common, particularly among oral surgeons and prosthodontists, whereas periodontists more frequently reported formal postgraduate training (p = 0.003). These findings highlight the differences in educational pathways despite comparable complication profiles (Tables 7, 8).

**Table 7:** Complications reported in the last two years (multiple response; n = 150 base). χ²: Pearson chi-square test, p > 0.05 indicated no statistical significance, data are presented as number (percentage), Q17 is a multiple-response variable with percentages calculated against the total sample size (n = 150), comparisons performed across specialties.

Complication type	Total n (%)	Periodontist, n (%)	Oral Surgeon, n (%)	Prosthodontist, n (%)	χ²	p-value
Graft necrosis	55 (36.7)	22 (33.8)	19 (42.2)	14 (35.0)	0.87	0.648
Severe postoperative bleeding	32 (21.3)	12 (18.5)	13 (28.9)	7 (17.5)	2.20	0.333
Infection	42 (28.0)	18 (27.7)	14 (31.1)	10 (25.0)	0.40	0.819
Patient dissatisfaction despite clinical success	62 (41.3)	26 (40.0)	18 (40.0)	18 (45.0)	0.30	0.859

**Table 8:** Primary training source for soft tissue augmentation around implants. χ²: Pearson chi-square test, *p < 0.05 indicates statistical significance, data are presented as number (percentage).

Training source	Total n (%)	Periodontist, n (%)	Oral Surgeon, n (%)	Prosthodontist, n (%)	χ²	p-value
Graduate program	28 (18.7)	18 (27.7)	5 (11.1)	5 (12.5)	19.72	0.003*
Postgraduate program	42 (28.0)	25 (38.5)	10 (22.2)	7 (17.5)		
Private training	52 (34.7)	12 (18.5)	22 (48.9)	18 (45.0)		
Continuing education course	28 (18.7)	10 (15.4)	8 (17.8)	10 (25.0)		

## Discussion

The present study provides insights into contemporary clinical decision-making, outcome perception, and failure attribution related to STA in implant dentistry. The findings revealed a combination of relative consensus in certain clinical thresholds alongside notable variability in technique selection, confidence levels, and perceptions of success and responsibility. A key observation was that most clinicians preferred to intervene at moderate levels of keratinized tissue width and buccal soft tissue thickness, rather than at extreme deficiencies. This suggests a pragmatic, experience-driven approach, rather than strict adherence to universally defined thresholds. The existing literature supports the importance of adequate peri-implant soft tissue dimensions in maintaining plaque control and minimizing inflammation, although definitive cutoff values remain controversial [2,6,8]. Studies by Fürhauser et al. [10] and Belser et al. [11] have highlighted that soft tissue characteristics significantly influence esthetic outcomes, reinforcing the clinical relevance of these parameters. The lack of significant interspecialty differences in threshold selection observed in the present study further suggests a broadly shared understanding of biological principles across disciplines.

Despite this agreement in thresholds, significant variation was noted in the technique selection, particularly in esthetically demanding scenarios. The preference for connective tissue grafts aligns with the current evidence that connective tissue grafts are the gold standard for increasing soft tissue thickness and achieving predictable outcomes [4]. However, the variability in choosing alternative approaches such as xenogeneic matrices reflects evolving clinical trends and the influence of training background and clinician preference. This is supported by systematic reviews indicating that soft-tissue substitutes can achieve outcomes comparable to autogenous grafts, although they may demonstrate less predictable results in terms of tissue stability and long-term gain [12].

The perception of failure rates in this study was generally low to moderate, consistent with the previously reported outcomes of STA procedures. However, an important finding was the inclination toward shared responsibility in cases of unexplained failure, shifting toward greater patient responsibility in the presence of risk factors, such as smoking [13,14]. This highlights an emerging awareness of the patient-related determinants of treatment outcomes. Although the literature on the economic and medicolegal aspects of implant therapy remains limited, these findings underscore the need for clearer guidelines regarding risk communication and consent.

Another significant observation was the discrepancy between subjective and objective evaluations of treatment success. While excellent outcomes are universally accepted, moderate outcomes are often deemed clinically acceptable but less suitable for academic reporting. This reflects the well-documented gap between clinical reality and publication standards, as discussed in esthetic indices, such as the pink esthetic score [9,10]. Such discrepancies emphasize the subjective nature of success in implant dentistry and the need for standardized outcome measures.

Confidence levels vary significantly across techniques and specialties, with higher confidence reported for connective tissue grafts and among periodontists for biomaterial use [4,15,16]. This likely reflects differences in training exposure, as supported by the finding that training pathways differ significantly among specialties. Previous studies have demonstrated that clinician experience and education strongly influence treatment choice and perceived competence [5]. In terms of complications, patient dissatisfaction despite clinically acceptable outcomes has emerged as the most common issue, highlighting the growing importance of patient-centered care and expectation management. This aligns with contemporary literature emphasizing that clinical success does not always equate to patient satisfaction, particularly in esthetic zones [17].

The present findings should also be interpreted within the context of the exploratory survey design and the subjective nature of clinician-reported perceptions. Several outcome domains assessed in this study, including failure attribution, confidence levels, and acceptable treatment outcomes, may be influenced by individual clinical experience, training background, practice setting, and regional healthcare systems. Furthermore, because multiple exploratory comparisons were performed, certain borderline statistically significant findings should be interpreted cautiously. The observed variability among clinicians nevertheless highlights the absence of universally accepted standards for evaluating success and failure in peri-implant soft tissue augmentation. Future multicenter studies incorporating standardized visual assessment tools, validated outcome criteria, and longitudinal clinical data may help improve reproducibility and strengthen evidence-based consensus in this area.

The clinical implications of this study include the need for more standardized guidelines for STA decision-making, improved calibration of outcome assessment, and enhanced communication with patients regarding risks and expectations. These findings also highlight the importance of structured training programs in reducing variability in practice. The present study has certain limitations that should be considered while interpreting the findings. The cross-sectional design and convenience sampling approach limit the ability to establish causal inferences and may affect the generalizability of the results beyond the surveyed population. Additionally, although the sample size was pragmatically determined for this exploratory survey, no formal calculation based on a predefined primary outcome or precision estimate was performed. The study relied on self-reported questionnaire responses, which may be influenced by response bias and recall bias, particularly for items related to perceived failure rates and complications experienced over the previous years. Furthermore, the use of hypothetical and verbally described clinical scenarios without standardized photographic references may have resulted in variability in interpretation among clinicians, especially for subjective soft tissue parameters such as contour, mucosal thickness, and esthetic asymmetry. The findings related to economic responsibility and medicolegal perceptions may also be influenced by regional healthcare systems, insurance structures, and local practice environments, thereby limiting broader applicability. Finally, several statistical comparisons were exploratory in nature and should therefore be interpreted cautiously.

## Conclusions

The present exploratory survey highlights variability in clinician decision-making, technique preference, outcome evaluation, and failure attribution related to soft tissue augmentation in implant dentistry. Although general agreement was observed regarding intervention thresholds and preference for connective tissue grafting, differences in confidence levels, training background, and perceptions of treatment success were evident among clinicians. The findings also demonstrate the influence of subjective interpretation and real-world professional experience on the assessment of soft tissue augmentation outcomes. Given the exploratory and region-specific nature of the study, the results should be interpreted cautiously and viewed primarily as hypothesis-generating. Nevertheless, the study provides preliminary insights into contemporary clinical perspectives in implant-related soft tissue management and supports the need for larger multicenter studies using standardized outcome assessment methods.

## Appendices

Appendix 1

**Table 9:** Questionnaire for the study. The questionnaire employed in this study was originally developed and validated by the authors.

Demographic details				
Name				
Age				
Gender	Male	Female		
Designation	Oral surgeon	Prosthodontist		Periodontist
Part 1- Practice Volume				
Q1- How many years have you been practicing implantology?				
<5	6-10	11-15	>15	
Q2- On average, how many dental implants do you place per month?				
<10	11-20	21-30		>30
Q3 In the last 12 months, how many soft tissue augmentation procedures around implants?				
None	1-5	6-10		>10
Part 2- Clinical Decision Thresholds				
Q4- What is the minimum keratinized tissue width (in mm) at a healthy implant that would prompt you to perform soft tissue augmentation, even in the absence of inflammation?				
<1 mm	1-2 mm	>2 mm		I do not augment based on KT alone
Q5- What is the minimum buccal soft tissue thickness (in mm) over an implant that would prompt you to augment, regardless of keratinized tissue?				
<1 mm	1-2 mm	>2 mm		I do not augment based on thickness alone
Q6- A patient with a single implant in the upper central incisor position presents with 0.5 mm keratinized tissue and 0.5 mm buccal thickness. The mucosa is thin and translucent. Which technique do you choose?				
No augmentation	Xenogeneic collagen matrix	CTG		FGG
Q7- The same patient as above, but now with a history of heavy smoking (>10 cigarettes/day) and poor oral hygiene. Would you change your technique?				
No, same technique	Yes, I would switch to a more invasive technique	Yes, I would avoid augmentation entirely		Yes, I would refer the patient
Part 3- Failure attribution & Economic risk				
Q8- In your experience, what is the approximate failure rate of soft tissue augmentation around implants (defined as ≥70% graft contraction or no gain in KT/thickness at 6 months)				
<5%	5-10%	11-20%		>20%
Q9- A CTG performed around an implant in the aesthetic zone fails (70% contraction) at 6 months. The patient had no obvious risk factors. Who should primarily bear the cost of revision surgery				
Patient	Clinician	Insurance		Shared 50/50
Q10- Would your answer to Q9 change if the patient was a known heavy smoker who denied cessation?				
No change	Yes, patient should bear 100% of cost	Yes, share 75% patient / 25% clinician		Yes, clinician bears less but still some responsibility
Q11- Have you ever been involved in a legal dispute or formal complaint related to soft tissue augmentation failure around an implant?				
Yes		No		
Part 4- Subjective vs. Objective success calibration				
Q12. Would you consider each of the following clinical scenarios a personal success (i.e., acceptable for you in daily practice)? (Please tick Yes or No for each scenario)				
Scenario 1 (Poor outcome): Graft contraction visible, thin mucosa, keratinized tissue width <1 mm	Yes	No		
Scenario 2 (Moderate outcome): 2 mm keratinized tissue width, 1.5 mm mucosal thickness, mild asymmetry	Yes	No		
Scenario 3 (Excellent outcome): >3 mm keratinized tissue width, >2 mm mucosal thickness, ideal contour	Yes	No		
Q13. Would you consider each of the same clinical scenarios publishable (i.e., acceptable to show in a peer-reviewed journal as a successful case)?				
Scenario 1 (Poor outcome as defined above)	Yes	No		
Scenario 2 (Moderate outcome as defined above)	Yes	No		
Scenario 3 (Excellent outcome as defined above)	Yes	No		
Part 5- Learning, Confidence & Complications				
Q14- How confident are you in performing soft tissue augmentation around implants using a connective tissue graft?				
Not confident	Slight confident	Moderate confident		Confident
Q15- How confident are you using a xenogeneic collagen matrix?				
Not confident	Slight confident	Moderate confident		Confident
Q16- In the last 2 years, have you experienced any of the following complications after soft tissue augmentation? (Select all that apply)				
Graft necrosis	Severe postoperative bleeding	Infections		Patient dissatisfaction despite clinical success
Q17- Where did you receive most of your training for soft tissue augmentation around implants?				
Graduate program	Post graduate program	Private training		Continuing education course
